# Supplementary material for: A sart1 Zebrafish Mutant Results in Developmental Defects in the Central Nervous System
Source: Cells. 2020 Oct 22;9(11):2340. doi: 10.3390/cells9112340 (PMC7690441; doi:10.3390/cells9112340)
Supplement: Supplementary file 1 [file cells-09-02340-s001.zip › Cells-959904-supplementary final-change/cellss-959904- supplementary-figure S1 and S2.pdf]

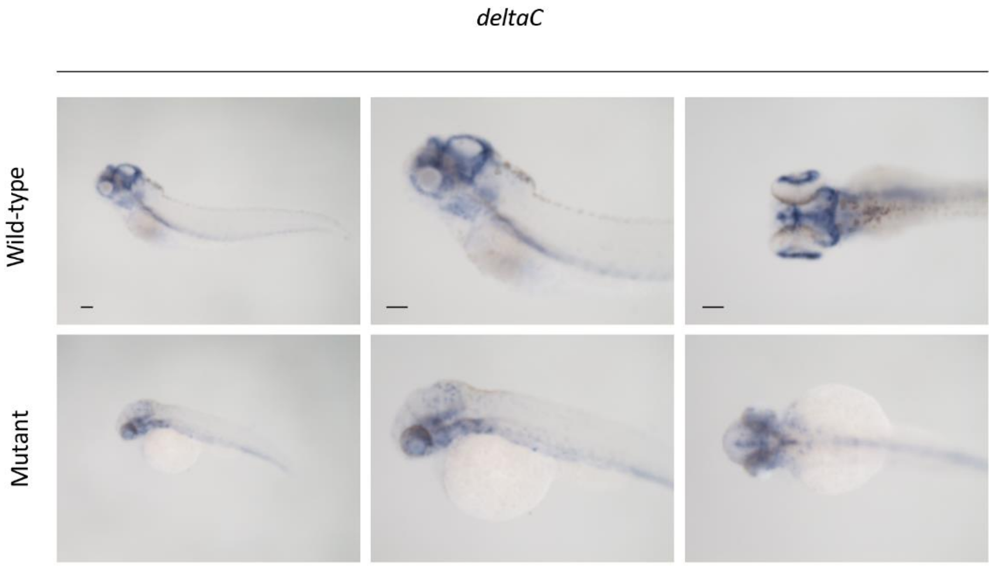

Figure 1. *deltaC* expression is disordered in *cp27.5* mutants. Whole mount in situ hybridization using *deltaC* as a control probe identified disordered expression in *cp27.5* mutants (bottom panel) compared to wild-type (top panel). In wild-type, *deltaC* is localized to the retina proliferative zone and brain ventricular zone. *deltaC* intensity is decreased in *cp27.5* mutant. While the probe does label the ventricular zone and eyes in mutants, the patterning is altered. Scale bars are 50 μm.

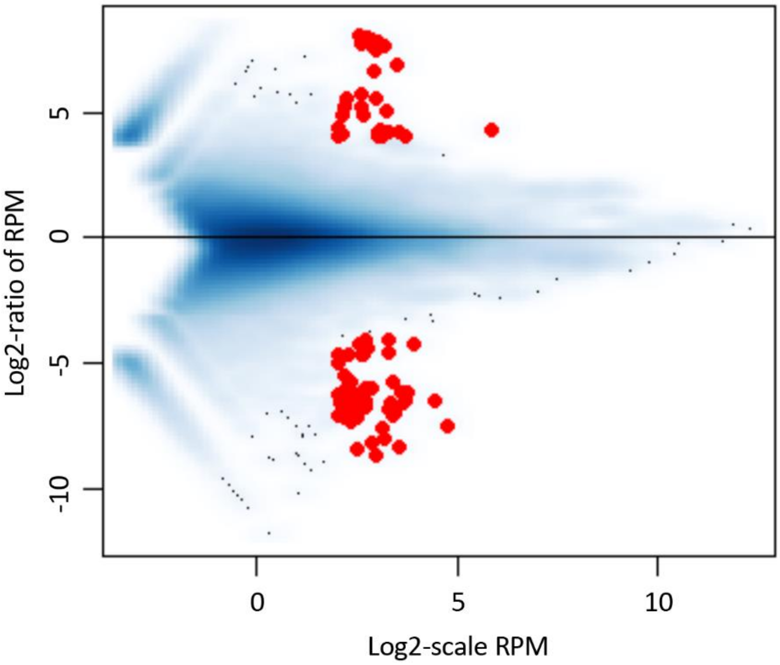

Figure 2. Scatter plot of log2 ratio of exon junction differential usage vs. log2 RPM of exon junctions with differentially used exon junctions in red. (RPM: reads per million).
