# Supplementary material for: A sart1 Zebrafish Mutant Results in Developmental Defects in the Central Nervous System
Source: Cells. 2020 Oct 22;9(11):2340. doi: 10.3390/cells9112340 (PMC7690441; doi:10.3390/cells9112340)
Supplement: Supplementary file 1 [file cells-09-02340-s001.zip › Cells-959904-supplementary final-change/supplementary FileS.pdf]

File S1: Dataset of RNA-Seq Analysis. RNA-Seq analysis identified a number of genes up regulated and down regulated due to a point mutation in sart1. This file contains the list of genes with at least one read observed in either one of the samples (wild type or mutant), genes expressed where the (FPKM > 0.1) in both samples, gene ontology for biological processes and molecular function, the Kyoto Encyclopedia of Genes and Genomes (KEGG), and a list of up regulated spliceosome-related genes.

File S2: Dataset of Novel Junctions for tp53 and mdm2. Analysis of tp53 and mdm2 identified novel splice junctions due to mutated sart1. This file contains a list of exon junctions for tp53 and mdm2 in wild-type and mutant samples. Candidate junctions were selected with a fold change of greater than 4 and an average RPM of the two samples that were more than 4. A description for each column in the data is as follows: ExonJunction: genomic location of the junction; Gene: gene name; Annotation_Set: the source for gene annotation; core: refseq; extended: ensemble; novel: does not overlap with known gene annotation; RefGene: refseq ID; EnsGene: ensemble gene ID; SJDRNORM010687_G2.27_5FXTL.WT.3dpf: read counts for the wild-type sample; SJDRNORM010965_G2.27_5FXTL.MUT.3dpf: read counts for the mutant sample; Wt:log2 read per million in SJDRNORM010687_G2.27_5FXTL.WT.3dpf; Mut: log2 read per million in SJDRNORM010965_G2.27_5FXTL.MUT.3dpf; average.intensity: average RPM of the two samples; log2r: log2 ratio between mutant and wild-type.
